# Cannabidiol (CBD) and Colorectal Tumorigenesis: Potential Dual Modulatory Roles via the Serotonergic Pathway

**DOI:** 10.3390/curroncol32070375

**Published:** 2025-06-26

**Authors:** Zhenhua Liu

**Affiliations:** 1 Nutrition and Cancer Prevention Laboratory, School of Public Health & Health Sciences, University of Massachusetts, Amherst, MA 01003, USA; zliu@nutrition.umass.edu; Tel.: +1-413-545-1075; 2 UMass Cancer Center, University of Massachusetts Chan Medical School, Worcester, MA 01605, USA

**Keywords:** cannabidiol, colorectal cancer, hemp, serotonergic pathway, *Wnt*/β-catenin pathway

## Abstract

The 2018 Farm Bill legalized hemp-derived cannabidiol (CBD) products containing less than 0.3% tetrahydrocannabinol (THC) in the United States. This legislative shift catalyzed both public and scientific interest in CBD’s potential health benefits. However, the rapid expansion of the CBD market has considerably outpaced rigorous scientific research, leaving many health claims largely unsubstantiated. While preclinical studies suggest that CBD may exert antitumorigenic effects in colorectal cancer (CRC) by modulating cell proliferation, apoptosis, and inflammation, clinical evidence supporting these effects remains limited. This review critically examines the current evidence on the role of CBD in colorectal tumorigenesis, with particular attention to its molecular mechanisms and interactions with the serotonergic system—a signaling pathway implicated in the development of CRC and possessing potential dual anti- and pro-tumorigenic properties. By influencing the serotonergic system, CBD may confer both protective and potentially deleterious effects during CRC development. This review underscores the need for further research to elucidate the complex mechanisms of CBD in colorectal tumorigenesis and to evaluate its therapeutic potential in clinical settings. Understanding these interactions could pave the way for novel prevention and treatment strategies, optimizing the anticancer efficacy of CBD while mitigating unintended risks.

## Simple Summary

Since hemp-derived cannabidiol products with less than 0.3% tetrahydrocannabinol became legal in 2018 in the United States, public interest in their health benefits has grown rapidly. However, scientific research has not kept pace, and many of the claimed benefits remain unproven. Early preclinical studies suggest that cannabidiol may help to combat colorectal cancer by influencing how cancer cells grow and die. One of the possible mechanisms is through its interaction with the body’s serotonergic system—a pathway that can have both helpful and harmful effects on cancer development. This review summarizes current scientific findings and emphasizes the need for more research to determine how cannabidiol works in the body and whether it is truly safe and effective for preventing or treating colorectal cancer. It offers important insights into the potentially dual effects of cannabidiol in the development of colorectal cancer amid its rapidly expanding use in health and wellness.

## 1. Introduction

The 2018 Farm Bill (the Agriculture Improvement Act of 2018, Pub. L. 115–334) was signed into law on 20 December 2018 [1], making the use of hemp-derived cannabidiol (CBD) products with less than 0.3% tetrahydrocannabinol (THC) federally legal in the United States [2,3]. Since then, public and scientific interest in CBD has surged exponentially, largely driven by a wide variety of potential health benefits purported to be due to its use [4,5,6,7,8]. However, the rapid growth of the CBD market has far exceeded the pace of scientific research, leading to numerous health benefit claims that lack rigorously scientific validation [3,9,10,11,12].

The most robust scientific evidence supporting the use of CBD pertains to its efficacy in treating certain severe forms of childhood epilepsy [13]. In the context of cancer, since the initial report in 1975 demonstrating the anticancer effects of cannabinoids [14], numerous studies have been conducted to investigate the potential of CBD, especially after the legislation in 2018 [8,15,16]. However, the use of CBD for cancer prevention and treatment requires further scientific investigation, and the specific tumorigenic pathways modulated by CBD remain largely undefined [17]. In this review, we aim to critically examine the potential effects of CBD, possibly both beneficial and adverse, on the development of colorectal cancer (CRC).

## 2. Cannabis, Cannabinoids, and Cannabidiol

Cannabis is a genus of flowering plants in the family *Cannabaceae* and is widely accepted as being indigenous to and originating from Asia [18,19,20,21]. The number of species within the genus is disputed, but *Cannabis sativa* is typically accepted as a single, undivided species. Cannabinoids are several structural classes of compounds found primarily in the cannabis plant [22]. Over a hundred distinct phytocannabinoids have been isolated from cannabis. The most notable cannabinoids are ∆9-tetrahydrocannabinol (THC), which is the primary psychoactive compound in cannabis, while cannabidiol (CBD) is a major constituent of cannabinoids from temperate cannabis plants and accounts for up to 40% of the plant’s extract [23,24,25].

### 2.1. Hemp vs. Marijuana

**Figure 1:**
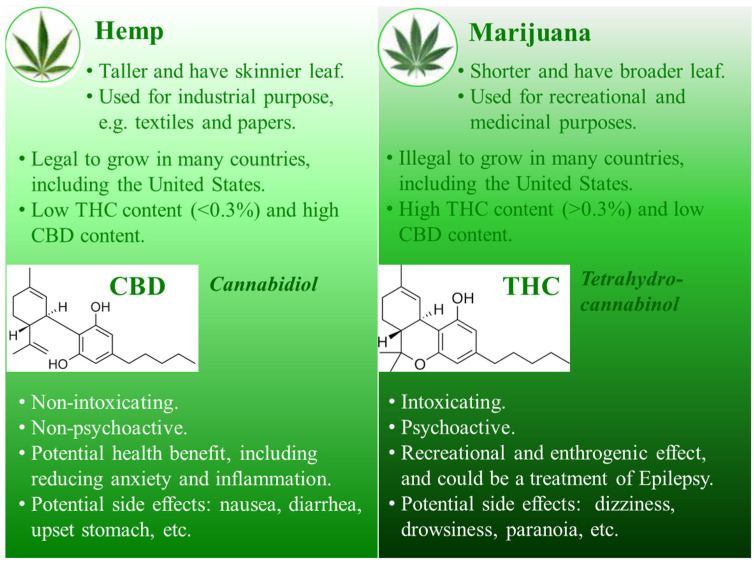
Differences between hemp and marijuana as well as the effects of cannabidiol (CBD) versus tetrahydrocannabinol (THC). Hemp and marijuana are not scientifically differentiated but are legally classified according to the content of THC. CBD is non-intoxicating with non-psychoactive effect, whereas THC is intoxicating and is psychoactive. CBD, cannabidiol; THC, tetrahydrocannabinol.

Hemp and marijuana plants are usually understood to be varieties of the same cannabis species, *Cannabis sativa* (Figure 1). While science does not differentiate between “hemp” and “marijuana” [26], the law does. Legally, hemp is defined as a cannabis plant that contains 0.3% or less THC, while marijuana is a cannabis plant that contains more than 0.3% THC [1]. This 0.3% cutoff value was first proposed in 1979 in a book called *The Species Problem in Cannabis: Science & Semantics* by Ernest Small [27], and the author acknowledged that the number is an arbitrary number, simply used to distinguish hemp from other cannabis strains with THC higher than 0.3% by dry weight.

Hemp, known as industrial cannabis, is among the fastest growing plants along with bamboo, is a plant cultivated specifically for industrial and consumable purposes, and can be used to produce a wide range of products (e.g., textiles, rope, clothing, biofuel, and animal feed) [28,29]. Hemp typically has lower concentrations of total THC but may have higher concentrations of CBD. Marijuana, also known as medical cannabis, has been used as a drug for both recreational and entheogenic purposes in various traditional medicines for centuries [30,31,32]. THC is the primary psychoactive compound in marijuana but the CBD content in marijuana is generally lower compared to that in the hemp plant.

### 2.2. Cannabidiol vs. Tetrahydrocannabinol

Cannabidiol (CBD) and tetrahydrocannabinol (THC) are similar in structure (Figure 1) but have very different effects: CBD does not produce psychotomimetic effects that are typically associated with THC [33,34]. Both CBD and THC have the same molecular formula—21 carbon atoms, 30 hydrogen atoms, and 2 oxygen atoms (C_21_H_30_O_2_, 314.5 g/mol) —but the way the atoms are arranged is different. CBD is a bicyclic compound whereas THC is tricyclic [35]. It is this slight difference in molecular structure that confers profoundly different pharmacological properties.

Despite both being cannabinoids and having the same chemical formula, the slightly different structural arrangement makes CBD and THC interact with different cannabinoid receptors in a person’s brain. The different interactions with cannabinoid receptors explain why THC is psychoactive while CBD is not. It is reported that THC can bind to cannabinoid receptor 1 (CB_1_), located in brain regions associated with emotions, learning, memory, movement, pain sensation, etc. [36]. This binding may account for its psychoactivity [37,38]. Unlike THC, CBD does not produce the high sensation typically associated with marijuana (high in THC) use; the research remains uncertain about the exact mechanism of CBD’s interaction with receptors, but experts believe that it binds differently from THC. A review [39] in 2018 reported that CBD may even reduce the ability of THC and other cannabinoids to bind to the cannabinoid receptors, and thereby reduce the psychoactive effects of THC and increase the number of circulating cannabinoids.

### 2.3. Legislation of Cannabis (CBD and THC) Use

Since CBD is not intoxicating and does not cause hyper psychoactivity, the 2018 Farm Bill removed hemp from the legal definition of marijuana in the Controlled Substances Act as mentioned in the Introduction section [1]. This made some hemp-derived CBD products with less than 0.3% THC federally legal. Since the legislation, there has been a rapid accumulation of evidence regarding the potential health benefits of CBD. An ever-growing body of preclinical and clinical research suggests that CBD may help to treat a variety of health conditions [40,41,42,43,44,45,46,47], though much of this evidence remains inclusive. In this review, we focus on evaluating the modulatory roles of CBD, potentially paradoxically, on the development of CRC.

## 3. Molecular Actions by Cannabidiol: Effects on Membrane Receptors

CBD exerts a wide spectrum of effects at the molecular and cellular level [47,48], responsible for its multiple therapeutic effects, including alleviating chronic pain [49,50], reducing symptoms of mental disorders such as depression, anxiety, and even psychosis [33,51,52], providing a neuroprotective effect [6,53,54], exerting anticancer properties [8,15,16], and even benefiting heart health [55,56]. Many of these actions are executed through its interactions with a variety of membrane receptors.

### 3.1. Interaction with the Endocannabinoid System

**Figure 2:**
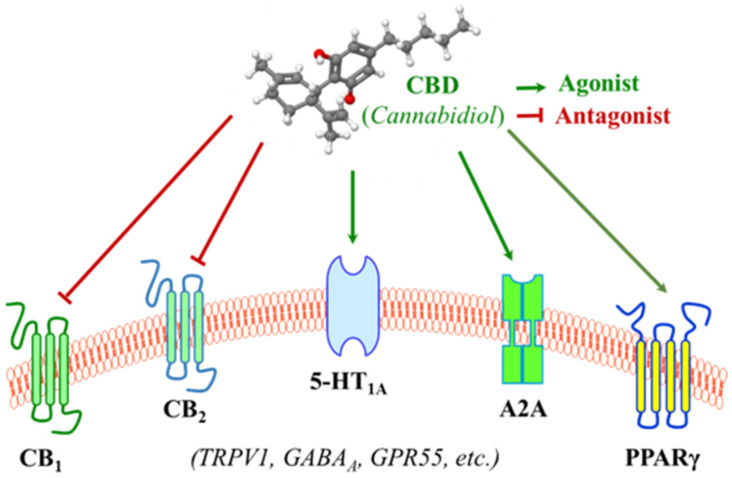
The diverse signaling targets of cannabidiol (CBD). CBD interacts with many receptors responsible for functions of the endocannabinoid system, the serotonergic system, and other signaling pathways. CB_1_ and CB_2_, cannabinoid receptor 1 and 2; 5-HT_1A_, 5-hydroxytryptamine (aka, serotonin) receptor 1A; A2A, adenosine receptor 2A; PPARγ, peroxisome proliferator-activated receptor gamma; TRPV1, transient receptor potential cation channel subfamily V1; GABA_A_, γ-aminobutyric acid type A; GPR55, G-protein-coupled receptor 55.

The most well-studied membrane receptors that CBD interacts with are cannabinoid receptor 1 and 2 (CB_1_ and CB_2_) (Figure 2) within the endocannabinoid system (ECS), a naturally biological system within humans that operates through signaling with endogenous cannabinoid molecules [10,57,58,59]. The ECS is composed of endocannabinoids, cannabinoid receptors, and enzymes responsible for cannabinoid synthesis and degradation [60,61]. The first cannabinoid receptor, CB_1_, was identified as a G-protein-coupled receptor derived from rat cerebral cortex cDNA in 1988 [62], and, in 1990, a protein homologous to CB_1_ was identified and was called CB_2_ [63]. CB_1_ and CB_2_ are two of the most notable receptors as they seem to be mainly responsible for endocannabinoid signaling [64]. CB_1_ is distributed across the central and peripheral nervous systems, with a notable presence on axon terminals in various regions of the brain, such as the cerebellum, hippocampus, hypothalamus, and midbrain [65]. CB_2_ is primarily expressed in peripheral tissue, notably on immune cells and intestinal epithelium, and plays a role in regulating cytokine productions and gut motility [66]. The exact mechanisms by which cannabinoids, including CBD and THC, exert their effects are not yet fully understood, but evidence notes that exogenous CBD and THC can bind to the cannabinoid receptors within the ECS as they have a similar chemical structure to anandamide, a naturally occurring endocannabinoid produced by the body [67,68]. However, as aforementioned in Section 2.2, CBD and THC may act on the cannabinoid receptors of the ECS differently due to their slightly but critically different structures.

At a functional level, THC exerts its function as an agonist via binding to both CB_1_ and CB_2_ in the ECS, though the binding to CB_2_ is relatively limited and less studied [69,70]. However, CBD has little binding affinity with CB_1_ and CB_2_, but it is capable of antagonizing them in the presence of THC and reducing the efficacy and potency of THC (Figure 2) [71,72]. CBD was originally isolated in 1940 and its structure and stereochemistry were determined in the 1960s [35]. Unlike THC as a direct agonist for cannabinoid receptors, it was originally proposed that CBD acts as a negative allosteric modulator of CB_1_ [71,73,74], and evidence also indicates that CBD functions as a CB_2_ receptor inverse agonist, contributing to its documented anti-inflammatory properties [71]. In summary, researchers do not yet fully understand how CBD interacts with the ECS, but it is known that CBD does not bind to CB_1_ or CB_2_ receptors in the same manner as THC, attributed to the lack of psychoactive properties that THC has.

### 3.2. Interaction with the Serotonergic System

Aside from its interactions with receptors in the ECS, the surge in research on CBD following the legislation in 2018 has uncovered numerous mechanisms involved in the large-spectrum therapeutic potentials of CBD, though many remain to be validated [25]. The serotonergic system is one of the oldest transmitter systems in the brain. A diversity of signaling opportunities and functional roles within this system explains the association of serotonin with many different types of psychopathological conditions [75]. However, most serotonin is found outside the central nervous system (CNS) and it regulates numerous biological processes other than neuropsychological processes [76]. The serotonergic system could contribute to the development of colorectal cancer (CRC) by modulating immune responses and DNA repair processes [77]. CBD may achieve this effect by interacting with serotonin receptors and modulating the downstream signaling pathways. For instance, CBD acts as an agonist of the 5-HT_1A_ serotonin receptor with a micromolar affinity (Figure 2) [78,79,80,81]. This interaction represents a connection between CBD and CRC, which is the primary focus of this review and will be delineated in greater details in later sections.

### 3.3. Interactions with Membrane Receptors Involved in Inflammatory Regulation

In addition to cannabinoid receptors and serotonin receptors, which play roles in the regulating of inflammatory signaling [82,83,84], CBD is also reported to modulate various other membrane receptors that are involved in immune cell function and the regulation of inflammatory cytokine productions (Figure 2) [85]. For instance, an in vivo treatment with a single and low dose of CBD resulted in decreased serum TNF-α levels in mice treated with lipopolysaccharide (LPS) as a consequence of the activation of the A2A adenosine receptors [86,87]. Another example is that CBD binds to and increases the transcriptional activity of PPARγ [88,89], which has an anti-inflammatory property in addition to its role in adipocyte differentiation, including the inhibition of pro-inflammatory cytokines IFNγ and TNFα [90,91]. These provide a non-cannabinoid receptor mechanism by which CBD can decrease inflammation.

In addition, it has also been reported that CBD interacts with several other membrane receptors (Figure 2). CBD activates vanilloid receptor type 1 (TRPV1) within the TRP channel, which is associated with its anti-inflammatory hyperalgesic property [92,93]. CBD is an allosteric modulator of gamma-aminobutyric acid type A (GABAA) receptors, the primary receptors for the neurotransmitter gamma-aminobutyric acid [94,95].

## 4. Cannabidiol in the Context of Colorectal Cancer

### 4.1. Colorectal Cancer Landscape

Colorectal cancer (CRC) remains a persistent public health challenge, impacting both men and women. Globally, CRC is the second leading cause of cancer-related death, with 903,859 deaths in 2022, accounting for 9.3% of all cancer deaths, and is the third most frequently diagnosed malignancy, preceded by lung and breast cancer, with 1,926,118 new cases in 2022, accounting for 9.6% of all new cancer diagnosed [96,97]. The World Health Organization (WHO) projected that, by 2040, the burden of CRC will increase to 3.2 million new cases and 1.6 million deaths per year [98]. In the United States, although the application of colonoscopy screening has reduced the incidence and mortality [99,100], CRC is still the third most common cancer and also the third leading cause of cancer death in both men and women, with a rate of ~5% in the lifetime. There have been ~150,000 new cases and ~50,000 deaths from CRC per year in recent years [101,102].

CRC is traditionally divided into sporadic and familial (hereditary) cases, and the proportion of familial CRC is 20–25% [103,104]. Among them, only ~5% of CRC is due directly to inherited genetic mutations [105]. Approximately 20% have a positive family history but cannot be categorized into having hereditary CRC syndrome [106,107]. The most common hereditary CRC are hereditary non-polyposis CRC (HNPCC) and familial adenomatous polyposis (FAP). HNPCC, accounting for 2–3% of all CRC cases [108], arises from microsatellite instability that is caused by the germline mutation of DNA mismatch repair genes [109]. FAP results from germline mutations in the adenomatous polyposis coli (*Apc*) gene, a key member in the *Wnt* pathway, but only accounts for <1% of all CRC [110,111,112]. Therefore, the majority of CRC is not the consequence of inherited genetic mutation but results from acquired defects under the influence of environmental or lifestyle factors, including unhealthy diets, alcohol consumption, smoking, physical inactivity, etc., and should be preventable [113].

### 4.2. Evidence of the Impact of CBD on Colorectal Tumorigenesis

Since the first report of the anticancer effects of cannabinoids, though it reported that CBD was active only in a high concentration [14], there have been many studies investigating its potential in the prevention and therapeutics of cancer [8,15,16]. Through an analysis of relevant studies from the biomedical databases Medline/PubMed, Scopus/Embase, Cochrane, and the Web of Science, we summarize some evidence on the use of CBD in the management of CRC (Table 1).

The information collected to date in relation to the anti-CRC effects of CBD is nearly completely limited to preclinical studies conducted on in vitro cell lines and in vivo animal models, without validation by clinical trials [16,136,137,138], while the impact of CBD on a variety of molecular targets, signaling pathways, and cancer hallmarks [139,140] has been observed. To date, a diverse array of colorectal carcinoma cell lines have been utilized to investigate the molecular mechanisms through which CBD exerts its effects (SW480 [114]; Caco-2 and HCT116 [115]; HCT116 and DLD-1 [116,118]; HCT116 [117]; HCT116, HT29, and DLD-1 [119]; oxaliplatin-resistant DLD-1 and colo205 [120]; HT-29 [122]; SW480 and HCT116 [123]; SW620, SW480, HCT116, and Caco-2 [124]; Caco-2 [125]; HCT116, SW620 and DLD-1 [126]; HCT116 p53wt vs. p53mut [127,128]; HT29, HCT116 and LS-174T [129]; HCT116 [130]; HT-29, SW480, HCT-116 and HCT-15 [131]; HT-29 and CCD 841 CoTr [132]; HCT116, HT-29, LS174T, and LS153 [135]). Through those in vitro cell culture studies, multiple CBD receptors were identified in CRC cell lines, acting either as agonists or antagonists. These receptors include CB_1_, CB_2_, TRPV1, PPARγ, and GPR55, among others. Several signaling pathways were implicated in response to CBD treatment, such as the promotion of ROS- and Noxa-dependent apoptotic signaling, inhibition of the Akt pathway, activation of the MAPK pathway, stimulation of autophagy, and suppression of *Wnt*/β-catenin signaling. These mechanisms collectively influenced several cancer hallmarks, like cell proliferation, cell viability/death, genome stability, cell metabolism, metastasis, and the immunosuppressive tumor microenvironment. Although the proposed mechanisms vary across studies and are not consistent, the majority demonstrated a protective effect of CBD against colorectal tumorigenesis.

The relatively limited in vivo animal studies have primarily focused on evaluating the impact of CBD on tumor development and tumor growth. In a chemical-induced CRC animal model (azoxymethane, AOM) [115,116], CBD reduced aberrant crypt foci (ACF), polyps, and tumors by counteracting AOM-induced Akt activation. In the HCT116 xenograft animal model [116,118,126], CBD inhibited tumor growth by upregulating the expression of Noxa and inducing apoptosis. In the colo205 xenograft animal model [120], CBD, in combination with oxaliplatin, inhibited tumor growth by decreasing p-NOS3 and SOD2 levels and inducing subsequent autophagy. In another xenograft animal model (xen: CT26) [121], CBD exerted an inhibitory effect on angiogenesis, tumor growth, and metastasis through reducing VEGF gene expression, decreasing cytokines, and increasing antioxidant enzyme activities. In a xenograft animal model with HCT116 p53wt or p53mut [127], it was demonstrated that a 5-week treatment with CBD reduced the tumor volume in a p53-dependent manner. In an MC38 xenograft C57BL/6 model [133], CBD reprogramed the metabolic process of macrophages, inhibited PI3K-Akt signaling, shaped the tumor microenvironment, and enhanced the response to anti-PD-1 immunotherapy to prevent tumor progression. In a dextran sulfate sodium (DSS)-induced colitis model [134], dietary CBD protected against inflammation and colitis symptoms via the activation of PKA/AMPK signaling.

## 5. Cannabidiol, Serotonin Pathway, and the Development of Colorectal Cancer

### 5.1. Serotonergic System in the Gastrointestinal Tract

Serotonin: Serotonin, also referred to as 5-hydroxytryptamine (5-HT), is a monoamine neurotransmitter and a regulatory hormone involved in a broad range of physiological processes [141,142]. Arguably, the well-known and classically defined function of 5-HT is to act as a neurotransmitter in the CNS, involving depression, anxiety, and happiness [143]. However, recent discoveries have significantly expanded our understanding of unconventional roles of peripheral serotonin within the gastrointestinal (GI) tract [144,145] and in a number of other tissues, such as the immune system [146]. In the GI tract, it acts as a hormone, with autocrine, paracrine, and endocrine functions. Although we are continuing to discover novel GI functions of 5-HT and how serotonin signaling is altered in GI disorders, the majority of its secrets remain to be revealed [145].

Although the traditional role of serotonin is the control of mood, only 1–2% of serotonin is produced by the neural cells in the CNS. Approximately 90% of the serotonin in the human body is produced in the GI tract [76], with an additional ~8% produced in other peripheral tissues. Serotonin is synthesized from the essential amino acid tryptophan by the rate-limiting enzyme tryptophan hydroxylase (TPH), for which there are two isoforms expressed in distinct cell types. *Tph1* is mainly expressed by specialized gut endocrine cells known as enterochromaffin cells (ECs) and by other non-neuronal cell types, such as adipocytes [147], and *Tph2* is primarily expressed in neurons in the brain and the enteric nervous system [148]. Since 5-HT cannot cross the blood–brain barrier, the central and peripheral pools of 5-HT are anatomically separated and thus function in their own distinct ways [149]. As excess serotonin within the body can also lead to toxic effects, an enzyme called monoamine oxidase, found mainly in platelets and enterocytes, can metabolize serotonin to reduce the amount of free serotonin in the body. Additionally, platelets and enterocytes express serotonin transporters (SERTs) that can absorb and sequester serotonin [148]. This is, in fact, the mechanism of action exploited by selective serotonin reuptake inhibitors (SSRIs).

Serotonin Receptors: Serotonin primarily exerts its effects through its receptors, and the impact that it has varies depending on the cells and tissues that express these receptors [150]. As of now, seven families of 5-HT receptors (5-HT_1_ through 5-HT_7_) have been identified. All of these receptors are G-protein-coupled, except for 5-HT_3_, which acts as a ligand-gated ion channel [151]. The receptors identified within these families so far include 5-HT_1(A, B, D, E, F)_; 5-HT_2(A, B, C)_; 5-HT_3(A, B, C, D, E)_; 5-HT_4_; 5-HT_5(A, B)_; 5-HT_6_; and 5-HT_7_. Since this review focuses on CRC, the discussion will center on receptors expressed in the gut (Table 2).

The function of serotonin in GI tract: Even though ECs constitute less than 1% of the total intestinal epithelium cells, they are located throughout the GI tract and produce >90% of the body’s 5-HT [163]. ECs release serotonin in response to food in the lumen, which makes the gut contract and increase intestinal motility. Serotonin modulates a wide range of physiological functions, including intestinal homeostasis. There are often serotonin abnormalities in gastrointestinal disorders such as celiac disease and irritable bowel syndrome [164,165]. Drugs, irritants, and toxins present in the food can stimulate ECs to release more serotonin and make the gut move faster, causing diarrhea. If serotonin is released in the blood faster than the platelets can absorb it, the increased level of free serotonin in the blood can activate serotonin receptors and stimulate vomiting [164,166].

### 5.2. Dual Effects of Serotonin in Colorectal Tumorigenesis

**Figure 3:**
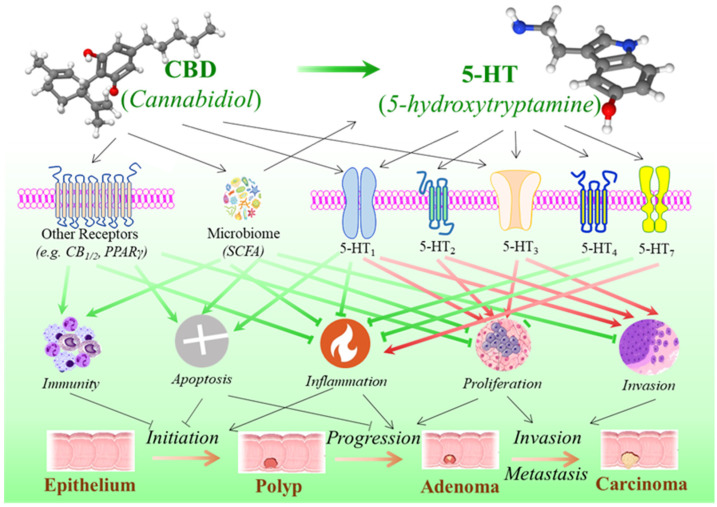
The impact of cannabidiol on serotonin pathway and the development of colorectal cancer. In addition to other molecular actions that cannabidiol may impose on the development of colorectal cancer, such as the endocannabinoid system, immune system, and microbiome, serotonin signaling represents one of the crucial pathways by which cannabidiol may have critical impact on the hallmarks of cancer. The impacts are complex and not yet to be fully delineated. It appears that cannabidiol may prevent tumor initiation in the early stages but promote progression and invasion/metastasis in the later stages of colorectal tumorigenesis.

Numerous studies have shown the functions of serotonin in the regulation of tumor biological processes like cell proliferation and apoptosis, invasion and metastasis, immunomodulation and inflammation, etc. It exerts its diverse, sometimes opposing actions through interaction with a variety of serotonin receptors linked to various tumorigenic signaling pathways [77,167]. Many studies show that serotonin has a potential stimulatory effect, especially in the later stage of the cancer process, including cell proliferation and migration, metastatic dissemination, and tumor angiogenesis [168,169], while emerging findings suggest that serotonin may play a dual role in CRC, where the impairment of the serotonin synthesis is linked with inflammatory reactions that may promote the initiation in colorectal tumorigenesis (Figure 3) [77].

Several serotonin receptor subtypes have been reported to be overexpressed in colorectal tumors or colorectal cancer cell lines, including 5-HT_1(B, D, F)_ [152,153], 5-HT_2B_ [156,157], 5-HT_3C_ [153], and 5-HT_4_ [153]. Evidence from in vitro cell culture studies has suggested that the blockade of the 5-HT_1B_ [155], 5-HT_2B_ [157], or 5-HT_3A_ [158] receptors, through either antagonists or gene knockdown, promotes apoptosis and reduces proliferation and migration. In contrast, receptor activation or overexpression has the opposite effect. In animal models, it was reported that overexpression of the 5-HT_1D_ receptor was associated with the *Wnt* signaling pathway and an advanced tumor stage, and its antagonist could effectively inhibit tumor metastasis through suppressing *Axin1*, a *Wnt*-signaling downstream gene [153]. A recent animal study demonstrated that 5-HT_2B_ is highly expressed in colorectal tumor tissues and that both the pharmacological inhibition and genetic knockdown of 5-HT_2B_ hindered the migration of CRC cells and disrupted the epithelial–mesenchymal transition process, indicating a regulatory role of 5-HT_2B_ signaling on CRC metastasis [156]. Silencing 5-HT_3A_ by short hairpin RNA slowed down tumor growth in an allograft CRC model, whereas treatment with the 5-HT_3A_ antagonist alleviated tumor progression in an azoxymethane/dextran sodium sulfate (AOM/DSS)-induced CRC mouse model [159].

Although studies mainly indicate that serotonin signaling through certain subtypes of 5-HT receptor families is linked with CRC cell proliferation, tumor growth, and metastasis, studies on animal models of intestinal colitis have shown that stimulating some of these serotonin receptors may help to prevent tumor initiation by reducing inflammation [170]. In an animal colitis model induced by 2,4,6-trinitrobenzenesulfonic acid (TNBS), the 5-HT_1A_ agonist delayed and mitigated the severity of colitis while the blockade of 5-HT_1A_ worsened it [154]. In both DSS- and TNBS-induced mouse models, 5-HT_4_ activation reduced inflammation in colons of mice with colitis, and, in noninflamed colons of wild-type mice, the inhibition of 5-HT_4_ resulted in decreased epithelial proliferation and barrier dysfunction and increased bacterial translocation to the liver and spleen, with signs of colitis within 3 days after the administration of 5-HT_4_ inhibitors [160]. In accurate DSS-induced and chronic IL-10 deficient colitis models, the pharmacological blockade or genetic ablation of 5-HT_7_ resulted in an increased severity of colitis, whereas receptor stimulation showed an anti-inflammatory effect [161], although another study contradictorily reported that the inhibition of 5-HT_7_ receptor signaling ameliorated both DSS- and TNBS-induced colitis [162]. In summary, while the molecular mechanisms of serotonin through its various receptors are highly complex and not yet fully understood, with inconsistent and even contradictory results to date, evidence from both in vitro and in vivo studies suggests a pattern where serotonin may have a dual role in CRC: it appears to prevent tumor initiation in the early stages but promote progression and invasion/metastasis in the later stages of colorectal tumorigenesis (Table 2 and Figure 3).

### 5.3. The Potential Influence of CBD on the Development of Colorectal Cancer via Serotonin Pathway

The direct evidence connecting CBD to the serotonergic system in the GI tract and colorectal cancer (CRC) is limited, but research indicates that CBD interacts with the serotonin pathway in other organs. CBD may influence the serotonergic system through three mechanisms (Figure 3): (***a***) growing research indicates that CBD modulates serotonin receptors. CBD is a strong agonist of the 5-HT_1A_ serotonin receptor, with a micromolar affinity. Acting as a partial agonist at this receptor, CBD can enhance serotonin signaling, which may contribute to its anxiolytic and antidepressant-like effects [78,79,80,81,171]. Compared to 5-HT_1A_, CBD exhibits a relatively lower affinity with 5-HT_2B_ [78]. In a *Xenopus laevis* oocyte model, data demonstrated that CBD acts as an allosteric inhibitor of 5-HT_3A_, and the inhibition of CBD was inversely correlated with 5-HT_3A_ expression levels [172]. (***b***) CBD may directly impact serotonin production by modulating tryptophan metabolism. An in vitro study on human peripheral blood mononuclear cells demonstrated that micromolar concentrations of CBD suppress tryptophan degradation, potentially increasing tryptophan availability for serotonin biosynthesis. This effect occurs independently of cannabinoid receptor activation. In contrast, nanomolar concentrations of CBD may enhance mitogen-induced tryptophan degradation in a CB_1_- or CB_2_-dependent manner [173,174]. (***c***) CBD may influence serotonin levels by modulating the gut microbiome. While further research is needed to fully elucidate CBD’s role in gut microbial dynamics, emerging evidence suggests that it influences gut health by altering the composition and diversity of gut bacteria [175,176]. The gut microbiome, in turn, has a bidirectional relationship with the host biosynthesis of serotonin [177,178,179,180], indicating a potential CBD–microbiome–serotonin connection.

As outlined in Section 5.2, serotonin may exert dual effects on colorectal tumorigenesis. A comprehensive review article [77] concluded that, while serotonergic activity may offer protection against early carcinogenic events in the colon, it could also contribute to CRC progression. CBD interacts with the serotonergic system, as previously mentioned, leading to speculation that it may have dual effects on colorectal tumorigenesis. While current evidence primarily supports a protective role of CBD in CRC (Table 1), further investigation into its potential dual effects at different stages of CRC could provide novel prevention or treatment strategies to maximize its anti-cancer efficacy while minimizing adverse effects.

## 6. Concluding Remarks

Since the legalization of hemp use in 2018, CBD has been promoted for a wide range of health benefits. While scientific evidence supports the effectiveness of CBD in treating childhood epilepsy syndromes, researchers still do not fully understand all the biological targets that CBD interacts with or its effectiveness on diseases other than epilepsy—the CBD boom has significantly outpaced scientific research. In the context of CRC, large-scale human studies comparing CBD with placebos are limited and much of the existing research has been conducted on cell culture or animal models, with findings that do not necessarily translate to humans. While current evidence from pre-clinical studies suggests that CBD has anti-tumor potential against CRC, this review explores the regulation of CBD on the serotonergic system and, consequently, the impact on colorectal tumorigenesis, highlighting the potential dual effects of CBD on the development of CRC. These insights into the relationship between CBD, the serotonergic pathway, and colorectal tumorigenesis underscores the urgent need for further research. Investigating the biological mechanisms and clinical benefits of CBD in CRC prevention and treatment is crucial to maximizing its therapeutic potential while mitigating any potential tumor-promoting effects.

## Figures and Tables

**Table 1:** Anti-colorectal cancer effects of cannabidiol as demonstrated in pre-clinical studies, using in vitro cell cultures or in vivo animal models.

References	Model	Dosage/Treatment	Molecular Actions	Hallmarks of Cancer
Sreevalsan et al., 2011 [114]	SW480	Up to 15 μM CBD	Dependent on CB_1_ on CB_2_; ↑Several phosphatases.	↓Cell proliferation; ↑Cell apoptosis.
Aviello et al., 2012 [115]	Caco-2 and HCT116	0.01–10 μM CBD	In a CB_1_-, TRPV1-, and PPARγ-antagonist manner.	↓Cell proliferation; ↓Genome mutation.
	Azoxymethane (AOM) CRC animal model	1 and 5 mg/kg CBD by injection	↑p-Akt (de-activation), NOS, COX2.	↓Aberrant crypt foci (ACF), polyps, and tumor.
Romano et al., 2014 [116]	HCT116 and DLD-1	1–5 μM CBD and CBD-BDS *	Dependent on CB_1_ and CB_2_.	↓Cell proliferation.
	Azoxymethane (AOM) CRC animal model	5 μM CBD-BDS	N/A	↓Aberrant crypt foci (ACF) and polyps.
	Male ICR mice (Xen: HCT116)	5 μM CBD-BDS	N/A	↓Tumor size.
Kargl et al., 2016 [117]	HCT116	1 or 2.5 μM CBD	Dependent on GPR55.	↓Adhesion and migration.
Jeong et al., 2019 [118]	HCT116 and DLD-1	6 μM CBD	Dependent on ROS and Noxa for apoptotic signaling.	↓Cell viability.
	BALB/c mice (Xen: HCT116)	10–20 mg/kg CBD by injection	Dependent on Noxa.	↓Tumor growth
Kim et al., 2019 [119]	HCT116, HT29, and DLD-1	4 μM CBD	↑CHOP, PERK, DR5.	↓Cell viability; ↑Cell apoptosis.
Jeong et al., 2019 [120]	Oxaliplatin-resistant DLD-1 and colo205	Up to 30 μM	↓p-NOS3, ↓NO production, ↑Autophagy; ↓SOD; ↑ROS production.	↓Cell proliferation; ↑Cell death.
	BALB/c mice (Xen: colo205)	10 mg/kg	↓p-NOS3; ↓SOD; ↑Autophagy.	↓Tumor growth.
Honarmand et al., 2019 [121]	BALB/c mice (Xen: CT26)	1–5 μM CBD	↓VEGF, IL-6 and IL-8; ↓Malondialdehyde; ↑SOD, GPx, GR.	↓Vasculature; ↓Tumor growth; ↓Metastasis.
Cerretani et al., 2020 [122]	HT-29	30 μM CBD	↑Malondialdehyde; ↓GPx, GR.	↓Cell viability.
Raup-Konsavage et al., 2020 [123]	SW480 and HCT116	10 μM pure CBD; 10 μM CBD oil **	N/A	↓Cell viability (pure CBD only).
Lee et al., 2022 [124]	SW620, SW480, HCT116, Caco-2	0–10 μM CBD	Dependent on CB_2_, but not CB_1_, TRPV, PPARγ; ↓Cyclin D1, Cyclin D3; ↓CDK2, CDK4, CDK6; ↑BiP, IRE1α, eIF2α, ATF3/4.	↓Cell viability; ↓Cell proliferation; ↑Cell apoptosis.
Nkune et al., 2022 [125]	Caco-2	1 μM	↑Photodamage.	↓Cell viability.
Feng et al., 2022 [126]	HCT116, SW620, and DLD-1	3, 6, 12 μM CBD	↓EMT; ↑E-cadherin; ↓N-cadherin, Snail, Vimentin, and HIF-1α; ↓Wnt-signaling.	↓Cell proliferation; ↓Cell migration; ↓Cell invasion.
	BALB/c mice (Xen: HCT116)	10 and 15 mg/kg CBD	N/A	↓Tumor volume.
Wang et al., 2023 [127], 2024 [128]	HCT116 p53wt vs. p53mut	5–20 μM CBD	Dependent on p53 and Hsp70; ↑ROS production; Trigger macroautophagy.	↓Cell viability.
	SCID mice (Xen: HCT116 p53wt or p53mut)	20 mg/kg CBD by injection	Dependent on p53.	↓Tumor growth.
Cherkasova et al., 2023 [129]	HT29, HCT116 and LS-174T	2–12 μM CBD	Altering TGF-β and MAPK signaling.	Altering cell metabolism.
Wei et al., 2024 [130]	HCT-116	N/A	↓SOD2/3 and ↑ROS; ↑Noxa; ↑Mitochondrial dysfunction.	↑Cancer cell death.
Kim et al., 2024 [131]	HT-29, SW480, HCT-116 and HCT-15	30 μM CBD	↑CHOP and ATF4; ↑ROS; ↑MAPK signaling; ↑Autophagy.	↑Apoptosis and paraptosis.
Paduch et al., 2024 [132]	HT-29 and CCD 841 CoTr	0–200 μg/mL	↓Mitochondrial dehydrogenase activity; ↓Nitric oxide.	↑Apoptosis.
Sun et al., 2024 [133]	C57BL/6 mice (Xen: MC38)	10 mg/kg CBD by injection	↓M2-like macrophages; ↑M1-like macrophages; ↓PI3K-Akt signaling.	↑Immune function.
Sun et al., 2024 [134]	DSS-induced colitis model	200 mg/kg CBD by dietary supplementation	↓Macrophage infiltration; ↑PKA/AMPK signaling; ↓NLRP3 inflammasome activation.	↓Disease activity index; ↓Inflammation and colitis symptoms.
Moniruzzaman et al., 2025 [135]	HCT116, HT-29, LS174T, and LS153	0–40 μg/mL	↑CB2 activation; ↑Endoplasmic reticulum (ER) stress.	↑Apoptosis; ↓Cell proliferation; ↓Cell migration; ↓Cell invasion.

* CBD-BDS: Cannabidiol botanical drug substances, which may also contain other cannabinoids at low levels as described by the authors [116]. ** CBD oil: extracted from hemp plants and diluted with carrier oil like coconut oil or hemp seed oil. The THC content was below 0.3% [123]. ↑: Increase or activation; ↓: decrease or inhibition. N/A: No data available yet.

**Table 2:** Summary of 5-HT receptor families 1 through 7 and their functions in the serotonergic system within the gastrointestinal tract.

Receptors	Gene Expression in GI ^1^		Functions in Gut	Expressions in Colorectal Tumor	Functions Related to Colorectal Tumorigenesis ^2^
	RNA	Protein			
5-HT_1(A, B, D, E, F)_	*Htr1a (low)*, *Htr1b*, *Htr1d*, *Htr1e (low)*	HTR1a, HTR1b (low), HTR1e	Intestinal motility; Immune protection.	↑5-HT_1(B, D, F)_ [152] ↑5-HT_1D_ [153]	↓/↑Inflammation and colitis (5-HT_1A_, A/AT) [154]; ↑CRC cell growth (5-HT_1B_, A) [155]; ↑Apoptosis, ↓Proliferation (5-HT_1B_, AT) [155]; ↑/↓*Wnt* signaling and metastasis (5-HT_1D_, A/AT) [153].
5-HT_2(A, B, C)_	*Htr2a (low)*, *Htr2b*	HTR2b	Intestinal motility; Intestinal secretion; Immune protection.	↑5-HT_2B_ [156,157]	↓Metastasis (5-HT_2B_, AT) [156]; ↓CRC cell growth (5-HT_2B_, AT) [157].
5-HT_3(A, B, C, D, E)_	*Htr3a*, *Htr3c (low)*, *Htr3e (low)*	HTR3a, HTR3e	Intestinal motility; Intestinal secretion; Immune protection.	↑5-HT_3C_ [153]	↑Apoptosis, ↓Proliferation and colony formation (5-HT_3A_, AT) [158]; ↑NLRP3 inflammasome (5-HT_3A_, A) [159]; ↓Tumor growth (5-HT_3A_, AT) [159].
5-HT_4_	*Htr4*	HTR4	Intestinal motility; Intestinal secretion; Immune protection.	↑5-HT_4_ [153]	↓/↑Inflammation and colitis (5-HT_4_, A) [160]; ↑Barrier dysfunction (5-HT_4_, AT) [160].
5-HT_5(A, B)_	*N/A*	HTR5a	N/A	N/A	N/A
5-HT_6_	*N/A*	N/A	N/A	N/A	N/A
5-HT_7_	*Htr7 (low)*	HTR7	Immune protection.	N/A	↑Inflammation and colitis (5-HT_7_, AT) [161]; ↓Inflammation and colitis (5-HT_7_, AT) [162].

^1^ The RNA and protein expression data are retrieved from the Human Protein Atlas (Version: 23.0, updated 19 June 2023). ^2^ ↑: Increase or activation; ↓: decrease or inhibition. A: Agonist or overexpression; AT: antagonist or knockdown. N/A: No data available yet.

## Abbreviations

TNBS	2,4,6-trinitrobenzenesulfonic acid
5-HT	5-hydroxytryptamine
ACF	Aberrant crypt foci
ATF3/4	Activating transcription factor 3, 4
APC	Adenomatous polyposis coli
A2A	Adenosine receptor 2A
MAPK	Amitogen-activated protein kinase
AOM/DSS	Azoxymethane/dextran sodium sulfate
CBD	Cannabidiol
CBD-BDSs	Cannabidiol botanical drug substances
CB1 and CB2	Cannabinoid receptor 1 and 2
CHOP	C/EBP homologous protein
CNS	Central nervous system
CRC	Colorectal cancer
CDK2/4/6	Cyclin-dependent kinase 2, 4, 6
COX-2	Cyclo-oxygenase-2
DR5	Death receptor 5
THC	D9-tetrahydrocannabinol
ECs	Enterochromaffin cells
EMT	Epithelial–mesenchymal transition
GPR55	G-protein-coupled receptor 55
GABAA	γ-aminobutyric acid type A receptor
GI	Gastrointestinal tract
GPx	Glutathione peroxidase
GR	Glutathione reductase
HIF-1α	Hypoxia-induced factor-1α
BiP	Immunoglobulin protein
IRE1α	Inositol-requiring enzyme 1α
NO	Nitric oxide
NOS3	Nitric oxide synthase 3
PPARγ	Peroxisome proliferator-activated receptor-gamma
eIF2α	Phosphorylated eukaryotic initiation factor 2α
PERK	Phosphorylated protein kinase RNA-like ER kinase
ROS	Reactive oxygen species
SSRIs	Serotonin reuptake inhibitors
SERTs	Serotonin transporters
SCFAs	Short-chain fatty acids
SOD	Superoxide dismutase
TGF-β	Transforming growth factor beta
TRPV	Transient receptor potential cation channel subfamily V
TPH	Tryptophan hydroxylase
VEGF	Vascular endothelial growth factor
